# Exogenous glucose oxidation during endurance exercise in hypoxia

**DOI:** 10.14814/phy2.14457

**Published:** 2020-07-11

**Authors:** Daichi Sumi, Nanako Hayashi, Haruka Yatsutani, Kazushige Goto

**Affiliations:** ^1^ Graduate School of Sports and Health Science Ritsumeikan University Kusatsu Shiga Japan; ^2^ Research Fellow of Japan Society for the Promotion of Science Chiyodaku Japan; ^3^ Faculty of Sports and Health Science Ritsumeikan University Kusatsu Shiga Japan

**Keywords:** endurance exercise, exogenous glucose oxidation, hypoxia, lactate

## Abstract

**Purpose:**

Endurance exercise in hypoxia promotes carbohydrate (CHO) metabolism. However, detailed CHO metabolism remains unclear. The purpose of this study was to evaluate the effects of endurance exercise in moderate hypoxia on exogenous glucose oxidation at the same energy expenditure or relative exercise intensity.

**Methods:**

Nine active healthy males completed three trials on different days, consisting of 30 min of running at each exercise intensity: (a) exercise at 65% of normoxic maximal oxygen uptake in normoxia [NOR, fraction of inspired oxygen (F_i_O_2_) = 20.9%, 10.6 ± 0.3 km/h], (b) exercise at the same relative exercise intensity with NOR in hypoxia (HYPR, F_i_O_2_ = 14.5%, 9.4 ± 0.3 km/h), and (c) exercise at the same absolute exercise intensity with NOR in hypoxia (HYPA, F_i_O_2_ = 14.5%, 10.6 ± 0.3 km/h). The subjects consumed ^113^C‐labeled glucose immediately before exercise, and expired gas samples were collected during exercise to determine ^13^C‐excretion (calculated by ^13^CO_2_/^12^CO_2_).

**Results:**

The exercise‐induced increase in blood lactate was significantly augmented in the HYPA than in the NOR and HYPR (*p* = .001). HYPA involved a significantly higher respiratory exchange ratio (RER) during exercise compared with the other two trials (*p* < .0001). In contrast, exogenous glucose oxidation (^13^C‐excretion) during exercise was significantly lower in the HYPA than in the NOR (*p* = .03). No significant differences were observed in blood lactate elevation, RER, or exogenous glucose oxidation between NOR and HYPR.

**Conclusion:**

Endurance exercise in moderate hypoxia caused a greater exercise‐induced blood lactate elevation and RER compared with the running exercise at same absolute exercise intensity in normoxia. However, exogenous glucose oxidation (^13^C‐excretion) during exercise was attenuated compared with the same exercise in normoxia.

## Graphical abstract

Exogenous glucose oxidation during endurace exercise in hypoxia was attenuates compared with same exercise in normoxia.

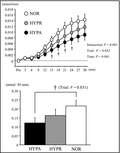

## 1 INTRODUCTION

The use of exercise training in normobaric hypoxia has been widely accepted as a potent tool for improving endurance capacity in athletes, and a large amount of experimental evidence supports the efficacy of this training method (Czuba et al., 2011, 2017; Dufour et al., 2006). Moreover, hypoxic training improves glycemic control in healthy people (Haufe, Wiesner, Engeli, Luft, & Jordan, 2008) and patients with type 2 diabetes or obesity (Chen, Lin, & Kuo, 2013; DE Groote et al., 2018; Mackenzie, Maxwell, Castle, Brickley, & Watt, 2011; Mackenzie et al., 2012). As a factor for the above benefit, endurance exercise in hypoxia augments carbohydrate (CHO) metabolism (e.g., increased CHO oxidation) compared with the same exercise in normoxia (Brooks et al., 1991; Katayama, Goto, Ishida, & Ogita, 2010; Morishima, Mori, Sasaki, & Goto, 2014; Sumi, Kojima, & Goto, 2018; Sumi, Kojima, Kasai, et al., 2018). Moreover, facilitated CHO mobilization (e.g., increased muscle glycogen utilization) during exercise in hypoxia would be a trigger for improving glycemic control after several weeks of hypoxic training. Previous studies (Katayama et al., 2010; Lecoultre, Boss, et al., 2010; Morishima et al., 2014; Sumi, Kojima, & Goto, 2018; Sumi, Kojima, Kasai, et al., 2018) have evaluated CHO metabolism during endurance exercise in hypoxia using several parameters including the respiratory exchange ratio (RER) and whole‐body substrate oxidation. However, the use of the RER may overestimate CHO oxidation during endurance exercise in hypoxia due to lower oxygen uptake and hyperventilation (with higher carbon dioxide output). In contrast, evaluating CHO oxidation using a stable isotope [^13^carbon (^13^C)] is expected to be an alternative procedure to overcome these problems (Harvey, Frew, Massicotte, Péronnet, & Rehrer, 2007; Lecoultre, Benoit, et al., 2010; Smith et al., 2010; Tremblay, Peronnet, Massicotte, & Lavoie, 2010). The consumed ^13^C‐labeled glucose is oxidized mainly in working muscles during exercise, and it is subsequently excreted into expired gas as ^13^CO_2_. Therefore, the breath ^13^CO_2_/^12^CO_2_ ratio during exercise reflects the amount of exogenous glucose oxidized. CHO metabolism evaluated by RER, blood lactate, and whole‐body substrate oxidation was enhanced in hypoxia (Katayama et al., 2010; Lecoultre, Boss, et al., 2010; Morishima et al., 2014; Sumi, Kojima, & Goto, 2018; Sumi, Kojima, Kasai, et al., 2018). Furthermore, exercise in hypoxia increases blood flow, which stimulates glucose delivery to the muscle and glucose disposal (Kjaer et al., 1999; Rowell, Saltin, Kiens, & Christensen, 1986). Therefore, exogenous glucose oxidation (^13^CO_2_/^12^CO_2_) would be increased in hypoxia.

A prevalent issue when comparing substrate oxidation during endurance exercise between normoxia and hypoxia is a difference in exercise intensity (absolute workload). Even when exercise intensity is relatively matched between hypoxia and normoxia [e.g., % of maximal oxygen uptake (VO_2max_) in each environment], absolute exercise intensity (e.g., running velocity or pedaling workload) is commonly lower in hypoxia due to decreased VO_2max_ in the hypoxia (Mollard, Woorons, Letournel, Cornolo, et al., 2007; Mollard, Woorons, Letournel, Lamberto, et al., 2007; Ofner et al., 2014; Wehrlin & Hallén, 2006). Therefore, the energy expenditure during endurance exercise is lower in hypoxia than in normoxia if the exercise is conducted at the same relative exercise intensity. Griffiths et al., (2019) demonstrated that CHO oxidation (calculated by the RER) during endurance exercise in hypoxia at moderate exercise intensity (50%–60% VO_2max_) was similar between hypoxia and normoxia when the same relative exercise intensity was used. In contrast, endurance exercise in hypoxia results in significantly higher CHO oxidation compared with the same exercise in normoxia when the same absolute exercise intensity is selected between normoxia and hypoxia. Considering that energy expenditure during endurance exercise strongly affects the amount of substrate oxidation, a comparison of CHO oxidation (hypoxia vs. normoxia) during endurance exercise at equivalent energy expenditure would be meaningful.

Therefore, the purpose of this study was to compare endurance exercise‐induced exogenous glucose oxidation between hypoxia and normoxia using both the same absolute exercise intensity and the same relative exercise intensity. We hypothesized that endurance exercise in hypoxia would augment ^13^C‐excretion (exogenous glucose oxidation) during exercise compared with the same exercise in normoxia, but that this difference would disappear when exercise intensity was relatively matched between normoxia and hypoxia.

## 2 MATERIALS AND METHODS

### 2.1 Subjects

Nine male subjects participated in this study. The number of subjects was determined based on the previous studies with determining physiological responses during endurance exercise in hypoxia (Katayama et al., 2010; Kelly & Basset, 2017; Sumi, Kasai, Ito, & Goto, 2019). The mean and standard error (SE) for age, height, and body mass were 24.4 ± 0.7 years, 175.3 ± 1.3 cm, and 68.2 ± 2.3 kg, respectively. All subjects were physically active with recreational resistance exercise or endurance exercise, but none were involved in regular resistance or endurance training within 6 months prior to the study. At time of testing, none of the participants were smokers, all subjects were injury free and not on any medications or dietary supplements. All subjects were born and living at sea level. They were informed of the experimental procedures and possible risks involved in this study, and informed consent was obtained. The study protocol was approved by the Ethics Committee for Human Experiments at Ritsumeikan University (BKC‐IRB‐2018‐003), and it was conducted in accordance with the Declaration of Helsinki.

### 2.2 Experimental design

Subjects visited the laboratory five times throughout the experimental period. During the first and second visits, two bouts of VO_2max_ testing were completed on a treadmill (Valiant; Lode, Groningen, the Netherlands) in either normoxia [inspired oxygen fraction (F_i_O_2_) = 20.9%) or normobaric hypoxia (F_i_O_2_ = 14.5%, equivalent to a simulated altitude of 3,000 m).

During the next three visits, the subjects ran for 30 min on a treadmill under one of three conditions: (a) 65% of normoxic VO_2max_ in normoxia [normoxia trial (NOR), F_i_O_2_ = 20.9%], (b) 65% of hypoxic VO_2max_ in hypoxia [hypoxia relative trial (HYPR), F_i_O_2_ = 14.5%], or (c) 65% of normoxic VO_2max_ in hypoxia [hypoxia absolute trial (HYPA), F_i_O_2_ = 14.5%] on different days. Therefore, relative exercise intensity (% of VO_2max_) was matched between NOR and HYPR. In contrast, NOR and HYPA used the same running velocity (absolute exercise intensity was matched). We used a cross‐over design, and each trial was separated by 1 week. Time‐course changes in blood variables and the ^13^C‐labeled carbon dioxide output ^13^CO_2_/^12^CO_2_ ratio in expired gas were monitored during exercise to clarify the effects of endurance exercise in hypoxia (trials with absolute or relative intensity matched for the same exercise in normoxia) on energy metabolism and exogenous glucose oxidation kinetics.

### 2.3 Exercise protocols

The subjects ran on a treadmill (Elevation series E95Ta; Life Fitness Corp.) in an environmentally controlled chamber during the three main trials. All trials were completed in an environmental chamber, and the normobaric hypoxic condition was established by insufflation of nitrogen. The subjects performed 30 min of continuous running exercise from 15 min after entering the hypoxic chamber using prescribed running velocities (NOR: 10.6 ± 0.3 km/hr, HYPR: 9.4 ± 0.3 km/hr, HYPA: 10.6 ± 0.3 km/hr) in hypoxia (for HYPR and HYPA) or normoxia (for NOR). Each trial was separated by 1 week. The three trials were started at the same time each day, and the order of the three trials was randomized. To avoid a psychological influence, subjects were not informed whether the trial was conducted in normoxia or hypoxia.

### 2.4 Measurements

#### 2.4.1 Maximal oxygen uptake (preliminary measurements)

Initial running velocity was set at 10 km/hr, and running velocity was increased by 2 km/hr every 2 min until 14 km/hr. Once running velocity reached 14 km/h, it was increased by 0.6 km/hr every 1 min until volitional exhaustion. The first criterion for exhaustion was maintenance of prescribed running velocity. In addition, we have confirmed all subjects met at least two of four criteria (VO_2_ plateau, respiratory exchange ratio > 1.10, HR of at least 90% of theoretical maximum, and rating of perceived exertion > 9 (10 scale) before determination of exhaustion. During the test, expired gases were collected and analyzed breath‐by‐breath using an automatic gas analyzer (AE300S; Minato Medical Science Co., Ltd., Tokyo, Japan). The data were averaged every 30 s. Heart rate (HR) was measured continuously during the test using a wireless HR monitor (Acculex Plus; Polar Electro Oy, Kempele, Finland). The VO_2max_ tests were performed twice in normoxia or hypoxia, and the order of the two repeated VO_2max_ tests was randomized. Each test was separated by three days.

#### 2.4.2 Exogenous glucose oxidation kinetics

Immediately before the onset of the exercise, the subjects consumed 500 mg ^13^C‐glucose (D‐Glucose‐U‐^13^C6, ^13^C: 99 atom %; Chlorella Industry Co., Ltd., Tokyo, Japan) dissolved in 100 ml purified water. The carbon atoms at all six positions in each glucose molecule were labeled with ^13^C. Before consuming the ^13^C‐glucose, a baseline breath sample was collected using a 1.3 L sampling bag (Otsuka Pharmaceutical Co., Ltd., Tokyo, Japan). A series of 10 breath samples were collected every 3 min during the 30 min exercise. The amount of ^13^CO_2_ in the sample bag was determined, and the ^13^CO_2_/^12^CO_2_ ratio was evaluated using an infrared spectrometer (POC one, Otsuka Pharmaceutical Co.). Changes in the ^13^CO_2_/^12^CO_2_ ratio were expressed as the absolute increase between samples during exercise and the sample at baseline.

The ^13^CO_2_ and ^12^CO_2_ abundance ratio was converted to the actual amount of excreted ^13^C, and then converted using the formula to evaluate ^13^C kinetics. ^13^C excretion per unit time was calculated using the following equation (Sumi et al., 2019; Tanaka et al., 2013),:

$$\text{Energy expenditure}\;\left(\text{kcal}/\min\right)=3.9\times {\dot{V}O}_{2}+1.1\times {\dot{V}\text{CO}}_{2}$$

The oxidation rates of carbohydrate and fat were calculated using the following equation (Manetta et al., 2002), where VO_2_ and VCO_2_ are in l/min; VO_2_ and VCO_2_ values were the averages of the last min during the 30‐min exercise:

$$\text{Carbohydrate}\left(g/\min\right)=4.585\times {\dot{V}\text{CO}}_{2}-3.226\times {\dot{V}O}_{2}$$

$$\text{Fat}\left(g/\min\right)=1.695\times {\dot{V}O}_{2}-1.701\times {\dot{V}\text{CO}}_{2}$$

#### 2.4.3 Rating of perceived exertion (RPE)

The subjects indicated their ratings of respiratory strain (RPE‐R) and leg strain (RPE‐L) at the end of the exercise using a 10‐point scale measuring perceived exertion (Wilson & Jones, 1991).

### 2.5 Statistical analyses

Data were expressed as mean ± standard error. A two‐way repeated‐measures analysis of variance (ANOVA) was used to test the interactions (trial × time) and main effects (trial, time). When ANOVA revealed a significant interaction or main effect, a Tukey–Kramer test was performed as a *post hoc* analysis to identify differences. The areas under the curve (AUC) for ^13^C‐excretion were compared between the three trials using a Tukey–Kramer test as a *post hoc* analysis to identify differences. For all tests, *p* values < .05 were considered significant.

## 3 RESULTS

### 3.1 VO_2max_, running velocity, and energy expenditure

VO_2max_ was significantly lower in hypoxia (42.6 ± 1.0 ml kg^−1^ min^−1^) than in normoxia (51.6 ± 1.1 ml kg^−1^ min^−1^, *p* < .0001). Consequently, running velocity during the 30 min of running (equivalent to 65% of VO_2max_) differed significantly among the trials (main effect for trial, *p* < .0001), and it was significantly lower in the HYPR than in the NOR and HYPA trials. However, no significant difference was observed between NOR and HYPA (Table 1). The energy expenditure during exercise differed significantly among trials (main effect for trial, *p* < .0001). HYPR involved significantly lower energy expenditure compared with NOR and HYPA. However, no significant difference in energy expenditure was observed between NOR and HYPA (Table 1).

**TABLE 1:** Running velocity and energy expenditure

	NOR	HYPR	HYPA
Running velocity (km/h)	10.6 ± 0.3	9.4 ± 0.3^a^	10.6 ± 0.3^b^
Energy expenditure (kcal/min)	11.7 ± 0.4	10.5 ± 0.4^a^	11.5 ± 0.3^b^

Note

Values are mean ± SE.

^a^ Significant difference versus NOR.

^b^ Significant difference versus HYPR

### 3.2 Exogenous glucose oxidation kinetics

**FIGURE 1:**
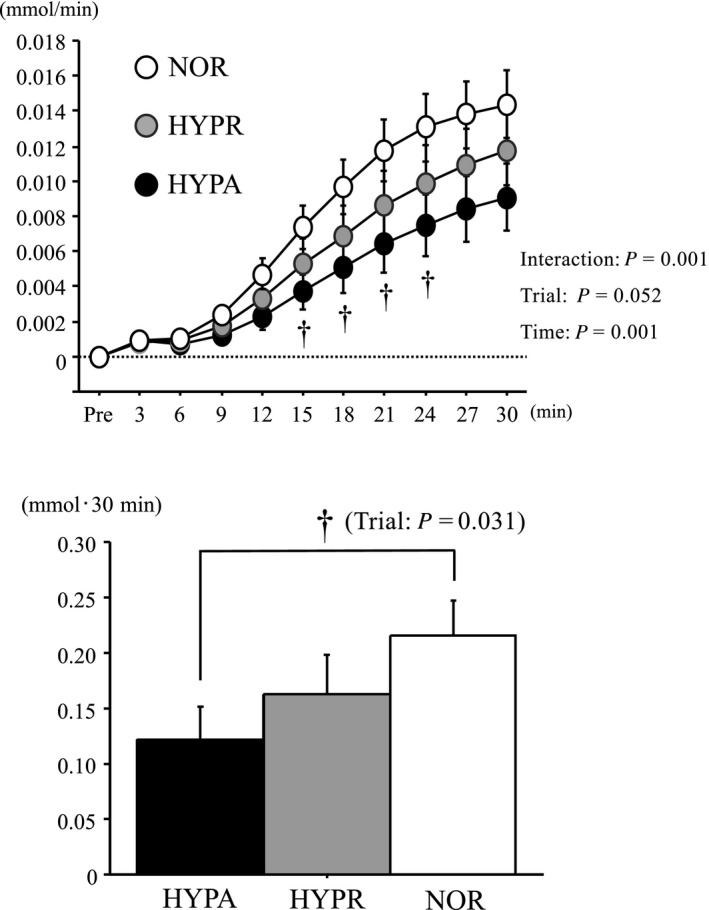
Changes in ^13^C‐excretion and area under the curve of ^13^C‐excretion during exercise. Values are means ± standard error (SE). †: Significant difference between NOR and HYPA

Figure 1 presents the changes in ^13^C‐excretion during exercise. ^13^C‐excretion, calculated as the absolute increase in ^13^CO_2_/^12^CO_2_ from baseline, increased during exercise in all trials (main effect for time, *p* < .001). However, HYPA involved significantly lower ^13^C‐excretion compared with NOR (interaction, *p* < .001, main effect for trial, *p* = .05). The AUC for ^13^C‐excretion was significantly lower in HYPA than in NOR (main effect for trials, *p* = .03). No significant difference was observed between HYPR and the other two trials.

### 3.3 Blood variables and plasma volume

**FIGURE 2:**
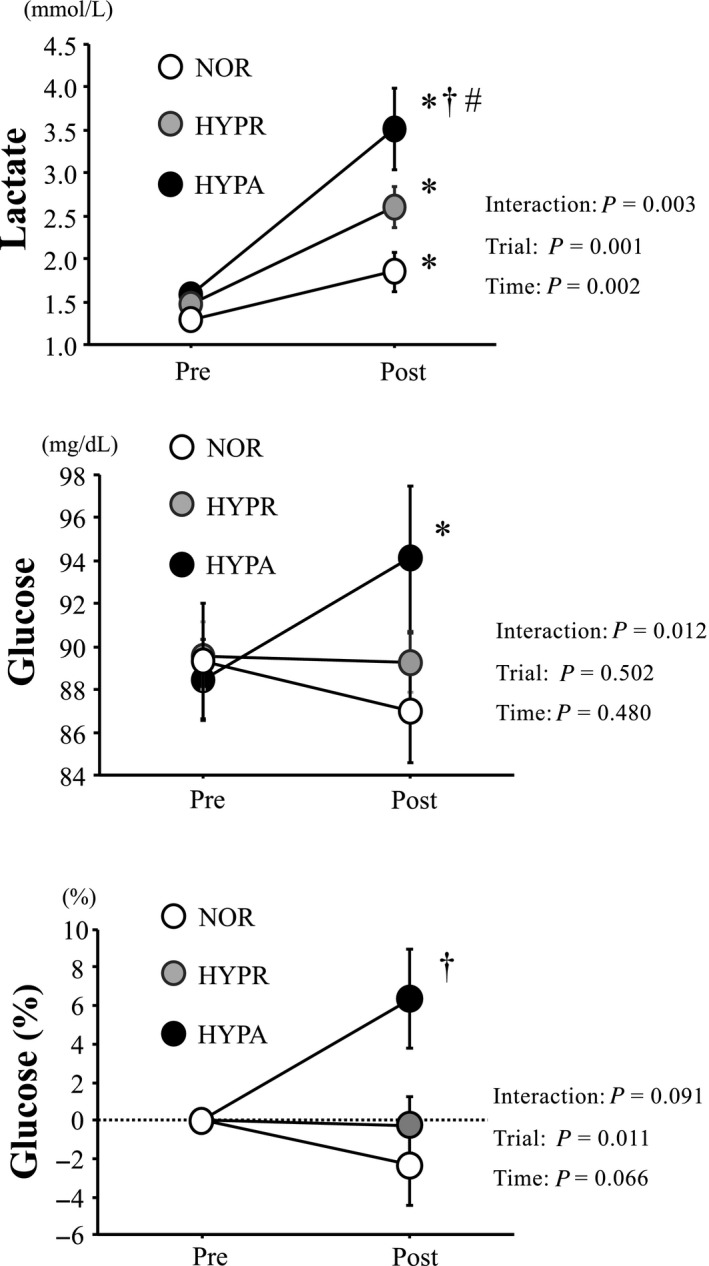
Changes in blood lactate and glucose concentrations. Values are means ± standard error (SE). *: Significant difference compared with pre‐exercise (Pre). †: Significant difference compared with NOR. #: Significant difference compared with HYPR

Figure 2 presents changes in blood lactate and glucose concentrations. Blood lactate concentration increased significantly after exercise in all trials (main effect for time, *p* = .002). Moreover, HYPA involved a significantly higher value than the NOR and HYPR trials after exercise (interaction, *p* = .003, main effect for trial, *p* = .001). Blood glucose concentrations increased significantly after exercise in HYPA (main effect for interaction, *p* = .01). However, no significant difference was detected among the three trials.

**FIGURE 3:**
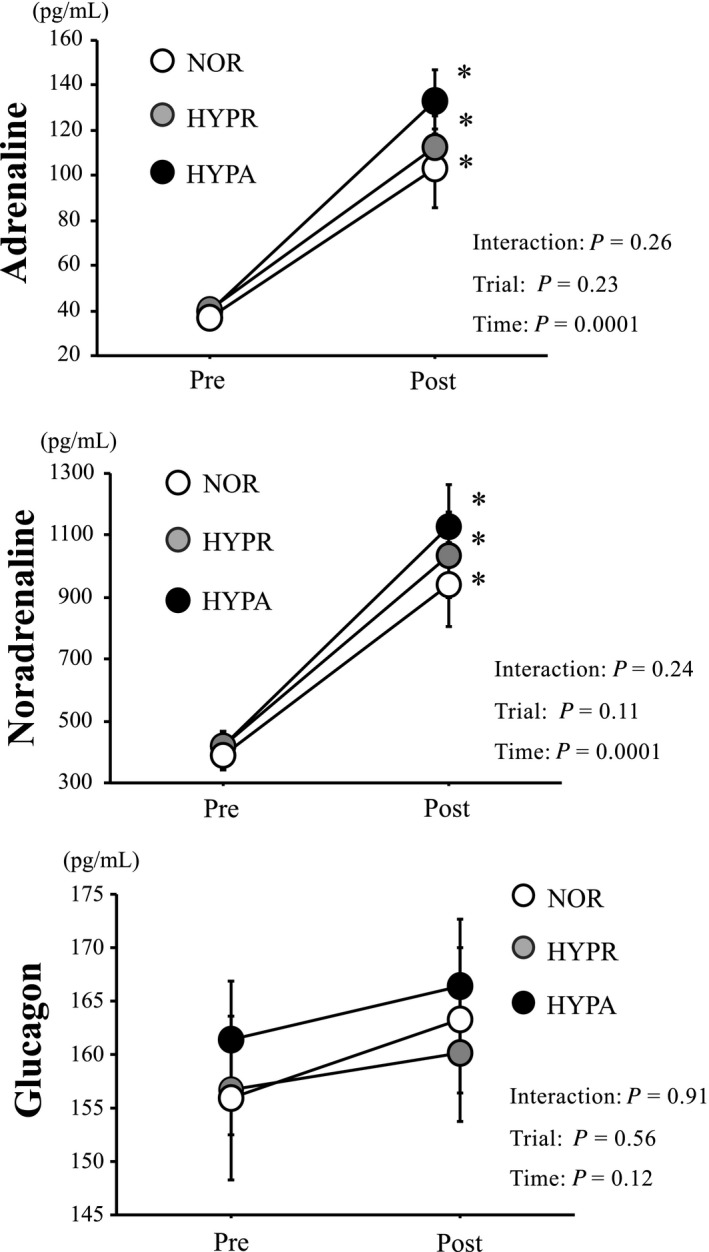
Changes in plasma adrenaline, noradrenaline, and glucagon concentrations. Values are means ± standard error (SE). *: Significant difference compared with pre‐exercise (Pre)

Figure 3 presents changes in plasma adrenaline, noradrenaline, and glucagon concentrations. Plasma adrenaline and noradrenaline concentrations increased significantly after exercise in the three trials (main effect for time, *p* = .0001). However, no significant difference was observed among the three trials. Plasma glucagon concentrations did not change significantly over time, and no significant difference was observed among the three trials.

Table 2 shows the changes in serum insulin, total ketone bodies, blood pH, HCO_3_^‐^, and plasma volume. Serum insulin concentration decreased significantly after exercise in NOR and HYPR (main effect for time, *p* = .001), but no significant difference was observed among the three trials. Serum total ketone body concentration did not change significantly after exercise. Moreover, no significant difference was observed among the three trials. Blood pH did not change significantly over time, and no significant difference was observed among the three trials. HCO_3_⁻ concentration decreased significantly in HYPA (main effect for time, *p* = .001). HYPA involved a significantly lower blood HCO_3_⁻ concentration immediately after exercise compared with the other two trials (interaction, *p* = .007). Plasma volume decreased significantly after exercise in the three trials (main effect for time, *p* = .004). However, no significant difference was observed among the three trials.

**TABLE 2:** Blood variables and plasma volume shift

	NOR		HYPR		HYPA	
	Pre	Post	Pre	Post	Pre	Post
Insulin (µlU/ml)	5.94 ± 0.9	2.10 ± 0.2^a^	5.66 ± 0.7	3.07 ± 0.4^a^	6.18 ± 0.9	3.76 ± 0.7
Ketone body (µnol/L)	47 ± 7.2	49 ± 7.6	63 ± 18.1	79 ± 23.9	52 ± 4.1	66 ± 7.2
pH	7.403 ± 0.01	7.419 ± 0.01	7.351 ± 0.05	7.425 ± 0.01	7.390 ± 0.01	7.408 ± 0.01
HCO_3_^−^(mmol/L)	28.1 ± 0.7	27.1 ± 0.6	26.2 ± 2.4	27.3 ± 0.6	28.2 ± 0.5	25.1 ± 0.5^a^, ^b^, ^c^
ΔPV (%)	0 ± 0	−5.1 ± 2.8^a^	0 ± 0	−5.1 ± 2.3^a^	0 ± 0	−7.6 ± 1.4^a^

Note

Values are mean ± SE.

Abbreviation: PV, plasma volume.

a Significant difference versus Pre.

^b^ Significant difference versus NOR.

^c^ Significant difference versus HYPR.

### 3.4 SpO_2_, cardiorespiratory variables, and substrate oxidation during exercise

Table 3 shows SpO_2_, cardiorespiratory variables, and substrate oxidation during exercise. The HYPR and HYPA involved significantly lower SpO_2_ compared with NOR (main effect for trials, *p* < .0001). However, no significant difference was observed between HYPR and HYPA. VO_2_ and VCO_2_ remained significantly lower in HYPR than in NOR and HYPA throughout the exercise (main effect for trials, *p* < .001 for each variables). No significant difference was observed in VO_2_, or VCO_2_ between NOR and HYPA. In contrast, HYPA involved a significantly higher RER (main effect for trials, *p* = .002), VE (main effect for trials, *p* < .0001), and HR (main effect for trials, *p* = .019) during exercise compared with NOR and HYPR. No significant difference was observed in the RER, VE, or HR between NOR and HYPR. CHO oxidation during exercise was significantly higher in HYPA (2.31 ± 0.1 g/min) than in NOR (1.79 ± 0.2 g/min) and HYPR (1.86 ± 0.1 g/min, main effect for trials, *p* = .008). In contrast, HYPA (0.30 ± 0.04 g/min) and HYPR (0.38 ± 0.03 g/min) involved significantly lower fat oxidation during exercise compared with NOR (0.54 ± 0.10 g/min, main effect for trials, *p* = .001). No significant difference was observed in fat oxidation between HYPR and HYPA.

**TABLE 3:** Cardiorespiratory variables and substrate oxidation during exercise

	NOR	HYPR	HYPA
SpO_2_ (%)	96 ± 0.5	82 ± 1.0^a^	82 ± 0.7^a^
VO_2_ (mL/kg min^−1^)	35.6 ± 0.6^b^	32.0 ± 0.7^a^	34.8 ± 0.7^b^
VCO_2_ (mL/kg min^−1^)	30.8 ± 0.8^b^	28.5 ± 0.9^a^	32.0 ± 1.0^b^
RER	0.86 ± 0.01	0.89 ± 0.01	0.92 ± 0.01^†^
VE (L/min)	65 ± 3.3	68 ± 2.1	81 ± 3.2^a^, ^b^
HR (beats/min)	156 ± 5.4	162 ± 4.4	170 ± 3.6
CHO oxidation (g/min)	1.79 ± 0.2	1.86 ± 0.1	2.31 ± 0.1^a^, ^b^
Fat oxidation (g/min)	0.54 ± 0.10^b^	0.38 ± 0.03^a^	0.30 ± 0.04^a^

Note

Values are mean ± SE.

^a^ Significant difference versus NOR.

^b^ Significant difference versus HYPR.

### 3.5 RPE

HYPA involved a significantly higher RPE‐L during exercise compared with NOR and HYPR (main effect for trials, *p* = .011). However, no significant difference was observed in RPE‐L between NOR and HYPR. The RPE‐R did not differ significantly during exercise among the three trials.

## 4 DISCUSSION

In this study, HYPA involved a significantly greater exercise‐induced blood lactate elevation and a higher RER compared with NOR. In contrast, the ^13^C‐excretion result was inconsistent, and it was significantly lower in HYPA than in NOR. However, these differences disappeared when exercise intensity was relatively matched between normoxia and hypoxia.

The exercise‐induced increases in blood lactate concentration and the RER were significantly augmented in HYPA compared with NOR, suggesting that endurance exercise in hypoxia promotes CHO metabolism compared with the same exercise in normoxia, which is consistent with findings from previous studies (Lundby & Van Hall, 2002; Parolin et al., 2000; Wadley et al., 2006). The present findings suggest that CHO metabolism during exercise in HYPA may be facilitated by increased ATP production via the anaerobic pathway (e.g., glycolysis) to compensate for the hypoxia‐induced decline in aerobic ATP production (Friedmann, Frese, Menold, & Bärtsch, 2007; Ogawa, Hayashi, Ichinose, Wada, & Nishiyasu, 2007).

The use of a stable isotope (^13^C) is a convenient procedure to evaluate CHO oxidation during exercise (Harvey et al., 2007; Lecoultre, Benoit, et al., 2010; Smith et al., 2010; Tremblay et al., 2010); specifically, exogenous glucose oxidation in the tissues (e.g., skeletal muscle and liver). Because the blood is predominantly distributed in working muscle during endurance exercise, an exercise‐induced increase in ^13^C‐excretion would mainly reflect glucose oxidation in working muscle. We also assessed changes in serum total ketone body concentrations as an indication of energy metabolism in the liver, but serum total ketone body concentration did not change significantly over time, suggesting that energy metabolism in the liver was not augmented.

Notably, the increase in blood lactate and the higher RER during endurance exercise were significantly greater in HYPA, whereas ^13^C‐excretion during exercise was attenuated compared with NOR. We did not expect this inconsistency, but the augmented blood lactate increase in HYPA was likely due to facilitated muscle glycogen utilization (augmented endogenous glycogen utilization) during exercise. Similar to the present findings, Jentjens, Wagenmakers, and Jeukendrup (2002) assessed the effect of endurance exercise in a hot environment on muscle glycogen utilization and exogenous glucose oxidation (evaluated by ^13^C‐labeled glucose). They found that a 90‐min cycling exercise at 55% maximal power output in a hot environment promoted muscle glycogen utilization compared with the same exercise in a neutral environment, whereas exogenous glucose oxidation (^13^C‐excretion) was attenuated, as shown in the HYPA in this study. Endurance exercise in hypoxia facilitates muscle glycogenolysis compared with exercise in normoxia when exercise intensity is matched absolutely between hypoxia and normoxia (Parolin et al., 2000; Wadley et al., 2006). Furthermore, augmented muscle glycogen utilization promotes the accumulation of glucose 6‐phosphate content in the muscle, which may inhibit glucose uptake into working muscle (Katz & Sahlin, 1989; Parolin et al., 2000). Furthermore, HYPA involved a significantly higher blood glucose concentration after exercise compared with NOR. Thus, we evaluated plasma glucagon and catecholamine responses as an indication of glycogenolytic response in the liver. However, no significant difference was observed in either plasma glucagon or catecholamine concentrations after exercise among the three trials. Therefore, higher blood glucose concentrations after exercise in the HYPA trial might be associated with lower glucose uptake in working muscle (i.e., leading to lower exogenous glucose oxidation) during exercise. The present findings suggest that the fuel dependence between blood glucose and muscle glycogen can be altered during endurance exercise in hypoxia, and the reliance of muscle glycogen for energy production may be augmented in hypoxia. Unfortunately, we could not evaluate the muscle glycogen content, which was a limitation in this study. Thus, future study is required to investigate the muscle glycogen utilization following an acute exercise session to clarify the detailed CHO metabolism during endurance exercise in hypoxia.

Many previous studies have determined energy metabolism during endurance exercise in hypoxia (Heinonen et al., 2012; Katayama et al., 2010; Kelly & Basset, 2017; Matu, Deighton, Ispoglou, & Duckworth, 2017; Wadley et al., 2006). However, consistent results have not been observed among related studies. One reason for this is the different exercise intensities between hypoxia and normoxia, because absolute exercise intensity (e.g., pedaling workload and running velocity) is generally lower in hypoxia than in normoxia when exercise intensity is relatively matched (e.g., % VO_2max_). The lower absolute exercise intensity in hypoxia is due to the reduction in VO_2max_ in hypoxia (Ofner et al., 2014; Sumi et al., 2019; Sumi, Kojima, & Goto, 2018; Sumi, Kojima, Kasai, et al., 2018). In this study, HYPA involved significantly different energy metabolism (i.e., blood lactate, ^13^C‐excretion and RER) from the NOR trial. In contrast, these differences were abolished between HYPR and NOR. Thus, the selection of exercise intensity (i.e., relatively matched intensity versus. absolutely matched intensity) and the difference in energy expenditure between hypoxia and normoxia strongly affect energy metabolism during endurance exercise.

Several factors are involved in the magnitude of exogenous glucose oxidation during exercise. For example, because the labeled glucose was orally ingested, gastric emptying and intestinal absorption of glucose affect exogenous glucose oxidation. Unfortunately, we were unable to assess the influence of exercise in hypoxia on gastric emptying and intestinal absorption due to a lack of ^13^C‐glucose enrichment data in the blood. However, no previous study has reported a negative (delayed) influence of hypoxia on gastric emptying and intestinal absorption. Future studies should focus on absorption of consumed glucose during endurance exercise in hypoxia to provide further information.

## 5 CONCLUSION

Endurance exercise in moderate hypoxia caused a greater exercise‐induced blood lactate elevation and RER compared with the exercise at the same absolute exercise intensity carried out in normoxia. However, exogenous glucose oxidation (^13^C‐excretion) during endurance exercise was attenuated compared with the same exercise in normoxia. Furthermore, these differences disappeared when exercise intensity was relatively matched between normoxia and hypoxia.

## CONFLICT OF INTEREST

The authors have no conflicts of interest, financial or otherwise, to declare.

## AUTHORS' CONTRIBUTIONS

DS contributed to the study design, data collection, analysis, and manuscript writing. NH contributed to the data collection, analysis. HY contributed to the study design, data collection, and analysis. KG contributed to the study design, data collection, analysis, and manuscript writing. All authors read and approved the final manuscript.
